# Medical school admission test: advantages for students whose parents are medical doctors?

**DOI:** 10.1186/s12909-015-0354-x

**Published:** 2015-04-23

**Authors:** Anne Simmenroth-Nayda, Yvonne Görlich

**Affiliations:** 1 Department of General Practice/Family Medicine, University Medical Center, Humboldtallee 38, 37073 Göttingen, Germany; 2 Study Deanery, University Medical Center, Humboldtallee 38, 37073 Göttingen, Germany

**Keywords:** Admission criteria, Medical school selection, Social background, Parents’ education, Socioeconomic factors

## Abstract

**Background:**

Admission candidates especially in medicine do not represent the socio-demographic proportions of the average population: children of parents with an academic background are highly overrepresented, and those with parents who are medical doctors represent quite a large and special group. At Göttingen University Medicine, a new admission procedure was established with the intention to broaden the base of applicants towards including candidates with previous medical training or lower final school grades. With a view to family background, we wished to know whether candidates differ in the test scores in our admission procedure.

**Methods:**

In February 2014 we asked all admission candidates of Göttingen University Medicine by questionnaire (nine closed, four open questions) about the academic background in their families, specifically, the medical background, school exam grades, and previous medical training as well as about how they prepared for the admission test. We also analysed data from admission scores of this group (semi-structured interview and four multiple mini-interviews). In addition to descriptive statistics, we used a Pearson correlation, means comparisons (t-test, analysis of variance), ANOVA, and a Scheffé test.

**Results:**

In February 2014 nearly half of the applicants (44%) at Göttingen University Medicine had a medical background, most frequently, their parents were physicians. This rate is much higher than reported in the literature. Other socio-demographic baseline data did not differ from the percentages given in the literature. Of all applicants, 20% had previous medical training. The group of applicants with parents who were medical doctors did not show any advantage in either test-scoring (MMI and interview), their individual preparation for the admission test, or in receiving or accepting a place at medical school. Candidates with parents who were medical doctors had scored slightly lower in school exam grades.

**Conclusion:**

Our results suggest that there is a self-selection bias as well as a pre-selection for this particular group of applicants. This effect has to be observed during future admission procedures.

## Background

Choosing the best-suited candidates for medical school is highly relevant for future patient care as well as for university and public resources. Medical schools aim at a fair, objective, and transparent admission process. It is also well established that ethnicity and social background may bias the outcomes of admission tests [1-4]. Moreover, admission candidates especially in medicine do not represent the socio-demographic proportions of the average population. Instead, students from upper social classes, notably from parents with an academic background, are highly overrepresented [5-9].

In Germany, this phenomenon has become even more pronounced over the past years: students in general tend to originate from families of the middle and upper classes, or in other words, from parents with an academic background. This is most frequently true for medical and law students. Between 65% and 74% of the medical students have parents with an academic background [10,11]. Another study [12] reported that 40% of the students had one parent and 25% both parents with an academic background.

Although there is still a debate of validity of several selection procedures, there is consensus today about objective admission-testing, which should include written entrance tests as well as structured interviews or multiple mini-interviews (MMI) that also assess communicative and social skills [13-18]. Göttingen University Medicine established a new admission procedure in 2013. The primary intention was to broaden the base of applicants, specifically to include candidates with a previous medical training or lower final school exam grades, which are more frequently present in students without academic family background. The first two rounds of the new procedure were attended by scientific monitoring including socio-demographic data and some questions about self-assessment and the degree of difficulty of the new procedure. When we realized that the first admission round consisted of very many students whose relatives held a medical degrees, we designed a more detailed socio-demografic questionnaire with the aim to quantify possible advantages of students with parents with medical background. We compared this with scores from the multiple MMIs and the semi-structured biographical interviews and also asked how this group individually prepared for our admission test. If this rather big group of applicants would have an advantage within our new procedure, we perhaps had to reconsider our concerns regarding more social admission criteria.

## Methods

### Context and admission-procedure

German law allows medical faculties themselves to select a quotum of 60% of the students [19]. Twenty percent of the applicants are chosen directly by best final school grade, the remaining 20% by waiting lists (if faculties miss this selection possibility, they in fact chose 80% of students by best school grades). If applicants wish to be chosen as part of the 60% quotum, they are asked to register with a national institution. This institution organizes the application procedures for all medical faculties in Germany [20]. It establishes a pre-selection (ranking list) by final school grades and grants a possible bonus for a completed professional medical training (among others: nurse, physiotherapist, occupational therapist). This is the first step of the admission procedure. At Göttingen University Medicine, we invite three times as many prospective students as we will be able to offer a place (within the 60% quotum). This procedure is repeated twice each year, and we see approximately the first 224 applicants from the then current ranking list. The second step consists of a 15-minute structured interview with two faculty members and four five-minute MMI-stations, where medicine-related situations (in part with simulated patients) and a self-reflecting case have to be passed. In the end, the final school grade is once again taken into account (which is mandatory: “predominant influence of the final school grade *in every step* of the admission procedure”). So the final school grade has a twofold impact within the whole admission procedure. Test quality and statistics from MMI and interviews are published elsewhere [21].

### Study design, participants

This is a quantitative questionnaire survey. We developed a questionnaire with nine closed and four open questions in summer 2013 and ran a pilot-study with the first round of the admission-test in August 2013. The questionnaire included items about difficulty and acceptance of MMI and interview (closed questions, 6-point lickert-scale) and the following open questions: 1) did you complete a medical training beforehand? If yes: which one? (free text) 2) did anyone in your family have a medical degree? If yes: who? (free text). 3) How did you prepared for the admission-test? (closed answers as well as free text). The questionnaire was then modified and finally used in February 2014 with the next admission round. Participants filled out the questionnaire voluntarily after passing the whole admission procedure. They were informed that participation would not influence the selection decision and gave written consent that the pseudonymized data were only to be used for research. The ethics committee informed us that only an “ethic request” without formal ethic-application was necessary and approved our study application number: 24/7/14An.

### Data management and statistics

We extracted data from two sources: 1) socio-demographic data including age, sex, and final school grades were submitted by the “Stiftung Hochschulstart”, a national central institution [20]. We 2) also generated our own data from the admission test itself: questionnaires (described above), test-scores of MMI, and score of the structured interviews. In addition to descriptive statistics, we used a Pearson correlation, means comparisons (t-test, analysis of variance), ANOVA, and a Scheffé test (SPSS-21).

### Ethical approval

The ethics committee informed us that only an “ethic request” without formal ethic-application was necessary and approved our study (application number: 24/7/14An).

## Results

Table 1 shows the characteristics of the participants. We collected complete data from 182 of 183 applicants. Nearly half of the applicants (44%) had a family background with some medical degrees. Twenty-four percent of the applicants with a family medical background had previous medical training. Because of European mobility it is also possible for applicants of other countries to apply at Göttingen Medical School. Only one applicant was accepted in university. Age, sex, country of birth and previous medical training did not differ for applicants with and without a family background. The properties of students who were offered a place at university and those who finally registered did not differ either.

**Table 1:** **Description of the participants**

Participants in summer 2014: n = 183	Physician in the family	No physician in the family		
**Participants who completed questionnaire: n = 182**	**80 (44%)**	**102 (56%)**	**p**	**test**
female (%)	58 (73)	77 (76)	0.65	chi-square
mean age (SD)	20,1 (2,18)	20,4 (2,21)	0.36	t-test
previous medical training* (%)	19 (24)	18 (18)	0.31	chi-square
final school exam abroad (%)	4 (5)	7 (7)	0.60	chi-square
accepted into university (%)	40 (50)	55 (54)	0.6	chi-square
registered in summer 2014 (%)	34 (85)	42 (76)	0.3	chi-square
**Participants accepted into university n = 95**	**n = 40 (42%)**	**n = 55 (58%)**		
female (%)	33 (83)	43 (78)	0.60	chi square
mean age (SD)	19,7 (2,0)	20,3 (2,49)	0.23	t-test
previous medical training* (%)	6 (15)	5 (9)	0.37	chi square
final school exam abroad (%)	1 (3)	2 (4)	0.76	chi square

*nurse, physiotherapist, occupational therapist, paramedic.

When a participant had given the information that there was some connection to medical jobs in his or her family, we asked for the specific relationship. Thirteen participants disclosed that both parents and 13 participants with both parents and additionally other relatives where physicians. When only one parent held a medical degree, it was most frequently the mother (11 versus 4 “only father”). In 23 cases, no parents, but several relatives had connections to medical work, in rare cases, only siblings were physicians.

As shown in Table 2, participants were divided into three groups: those where one parent was a physician, those with a physician amongst their other relatives, and those without physicians in their families. Table 2 shows that there is no significant difference in the scores of the three groups. Scores in interview and MMI-stations were distributed normally. The highest point score at the MMI-stations was 5, the highest interview point score was 10 (two raters, each 5 points at most). The final school grade was converted into 31 points because of statutory rules (“predominant influence of school grades”), which means that the final admission-score was 61 at most. The only indication of a difference was found in the final school examination grades, where candidates with physicians as parents had scored slightly lower; this difference was also significant according to the ANOVA test, but not according to the Scheffé test, which is a post-hoc test (p = 0.06).

**Table 2:** **One-way ANOVA admission-test scores and final school grade**

Summer semester 2014		One or both parents are a physician n = 50 (44%)	Other relatives are physicians n = 102 (56%)	No physician in the family n = 102 (56%)	p-value**
Assessed dimension in admission test	Max. score	Mean score (SD)	Mean score (SD)	Mean score (SD)	
interview: motivation and potential	10	7.03 (1.37)	7.01 (1.71)	7.00 (1.47)	0.991
MMI-1: counseling skills and spontaneity	5	3.32 (0.75)	3.16 (0.91)	3.30 (0.78)	0.648
MMI-2: verbal skills and analytic skills	5	3.69 (0.93)	3.34 (1.08)	3.50 (1.00)	0.302
MMI-3: stress resistance and social competence	5	3.74 (0.67)	3.74 (0.56)	3.75 (0.64)	0.992
MMI-4: empathy and social competence	5	3.29 (0.89)	3.44 (0.83)	3.22 (0.89)	0,481
**sum-scores**					
score interview and MMI	30	21.06 (2.76)	20.69 (3.23)	20.77 (3.12)	0.825
score final school grade*	31	25.92 (1.50)	26.70 (1.74)	26.52 (1.58)	**0.047**
total score (admission test plus final school grade)	61	46.98 (2.88)	47.39 (2.99)	47.29 (3.13)	0.794

*translated from max. 15 points in German final school exam grade. **scores that show significant differences between the groups are printed in bold.

We asked the participants how they prepared for the admission test. We found a slight difference in the field of “talking with family and friends” in favors of the group that had a “family background with medical jobs”. Only one applicant of this group visited a special preliminary course (Table 3).

**Table 3:** **How did you prepare for the admission test?**

Summer semester 2014	n = 182	Physician in the family n = 80 (44%)	No physician in the family n = 102 (56%)		
Item (multiple answers allowed)		n (%)	n (%)	Effect size	p-value**
information via internet or internet forums		67 (84%)	90 (88%)	0.13	0.386
talked with family and friends		53 (66%)	49 (48%)	0.37	**0.014**
with books or brochures		13 (16%)	20 (20%)	0.09	0.562
others* (written answers)		13 (16%)	18 (18%)	0.04	0.805
with specific training or courses		1 (1%)	0 (0%)	0.16	0.260

*talking with other physicians, nurses, or medical students, reflecting actual medical news in TV or newspapers, self-reflection about career choice, simulated interview. **scores that show significant differences between the groups are printed in bold.

## Discussion

In summer 2013, nearly half of the applicants at Göttingen University Medicine had a family background that included family members with medical degrees, most frequently, parents were physicians. In our admission procedure, this special group did not score any advantage in either test-scoring or individual preparation for the admission test itself, but apparently in choosing our admission procedure as such.

### Strength and limitations

We worked with complete and high-quality data: all but one applicant filled out the questionnaire with the socio-demographic data. The reason for this high quote is perhaps the high motivation on the part of the applicants, who were invited to our new selection procedure in spite of a lower final school grade. After filling out the questionnaire, many applicants spontaneously gave additional oral feedback (“I am so happy to be here and to have this chance”, “I think this is a really fair procedure that includes other skills, not only final school grades”). Of course we cannot guarantee that some applicants felt pressure to fill the questionnaire, because they wanted to make a good impression in this application situation.

Socio-demographic data are accurate, because they are center-fed from the national authority [20]: when applicants have to register, they are asked to give truthfully information (e.g. scan of officially certified final school exam grade, declaration on the accuracy of all data). MMI test scores and interview scores are also valid because the procedure at Göttingen University Medicine has been legally approved. The high proportion of applicants with previous medical training can probably be explained by the bonus, which is an upgrading of the final school grade that these applicants receive. The high rate of female applicants is due to the influence the final school grade has on the whole procedure: the proportion of schoolgirls with best final school exam grades is much higher than of the boys, therefore the chance to be invited to our admission procedure is much higher. This phenomenon has also been observed in Germany and other countries in the past years [22].

Unfortunately, we did not ask about the highest level of the parents’ degrees in general, therefore we were unable to compare our data with the situation of students’ parents in Germany in general. Our proportion of 44% applicants with a family background with medical degrees (33% had two medical doctors as parents, 66% had one medical doctor as parent) is even higher than reported in Germany or internationally [7,8,10,11]. Some possible explanations are that the procedure is relatively new at Göttingen University Medicine, it was established in 2014, and the good medial visibility on the university homepage and in medical local journals, which may have caused an information advantage for medical colleagues and their children. This last is only a hypothesis and has to be examined with future multicenter applicant rounds. We do not know the proportion of students with medical doctors as parents in our older students at Göttingen University Medicine. They matriculated under other conditions (we only asked about the final school grade and whether they had any previous medical training), therefore we are unable to determine whether this is a local or temporal phenomenon.

### Comparison with the literature

Students and applicants with parents who hold a medical degree represent a very special group: a Norwegian study reported a proportion of 12% of physician children in medical schools; this number was stable over many years. Likewise, 24% of the Danish medical students, interviewed between 2005 and 2007, had parents with academic degrees and 16% had parents who were medical doctors [6,8]. In Australia, 57% of applicants had parents with a medical background, which was not further specified into academic or other medical professions. Most students with parents with a medical degree in general are not required to contribute as much to their own expenses because their parents hold well-paid jobs. Thus the students have more time and resources for their own training and to prepare for the tests. Finally, candidates with an academic background tend to be more familiar with academic rules, communication, and behaviour [6,23-25]. Perhaps complex admission procedures for applicants from non-academic households are more daunting than presumably objective school exam grades, while they are in contrast more familiar to those living in a medical household. This greater affinity to and familiarity with academic surroundings of students who grew up in a family with a medical background is also documented by the information that 13% of the medical students in Germany attested that they expect to inherit the medical practice from one of their parents, which is possible in Germany because of the self-employed status of nearly all ambulant health-care providers [10]. This means that a strong identification with the parental profession can be assumed. Perhaps this is also the reason for the relatively high proportion of medical trained applicants.

To our knowledge, no data exists about admission-test scoring especially from this particular group of applicants in Germany. There are also few international data about children of physicians and their possibilities and skills in acquiring a place at university for medicine, and no data at all about admission-scorings in detail [6,8,26]. In Denmark, O’Neill [27] reported that the drop-out rates of medical students with parents with medical degrees were the same as those of other medical students. It can be assumed that in admission tests there would be no difference to other applicants in this group either.

Despite the equal success of applicants with a family background in medicine and of applicants without this, the access to admission procedures should be possible and transparent for all, regardless of their social background. More diversity within professional groups is desirable, especially among physicians, who work with patients who hold a broad range of professions, and thus the physicians may need a broader background to understand their patients’ needs [26,28]. A former medical training as such may help to communicate with patients and to have a deeper insight in patients perspective: students with former medical training show better communicative and social skills and are also more successful in passing medical school [29,30]. One new possibility to promote this idea in Germany is the permission for a few applicants to enter medical school without a baccalaureate, but with an equivalent to a master certificate from medical-related professions [19].

### Preparation and information about the admission test

The internet is by far the most frequently accessed information source today. Special preparations for admission tests are common in Germany, as in other countries, but results about the effects of these preparations are inconsistent [31,32]. In one case, applicants who attended coaching for MMI did not perform better than applicants without coaching [33], while Laurence et al. [29] described the opposite effect. As Laurence reported, more than 50% of applicants with a medical background in our group talked about the admission test with relatives, but without later benefiting in the scoring. We assume that relatives can probably better inform about the general setup of the studies and the final profession, but not about content details of admission procedures. Applicants without a medical background or with little contact to the academic world perhaps face an emotional and financial hurdle in preparing for special academic admission tests and also for applying ultimately [24].

## Conclusion

Nearly half of our applicants in our new admission procedure had doctor parents, similar trends are known from other professions and were socially accepted in ancient times. More research should be done about this phenomenon today in the medical context. Our results suggest that there is a self-selection bias as well as a pre-selection for applicants with doctor parents, but results in scoring did not differ from those of the other applicants. We are curious about the following rounds of applicants and the academic biographies of the current students, which we wish to observe carefully from now on.
